# First-Episode of Synthetic Cannabinoid-Induced Psychosis in a Young Adult, Successfully Managed with Hospitalization and Risperidone

**DOI:** 10.1155/2016/7257489

**Published:** 2016-06-26

**Authors:** Aaron J. Roberto, Aileen Lorenzo, Kevin J. Li, Jonathan Young, Abhishek Mohan, Subhash Pinnaka, Kyle A. B. Lapidus

**Affiliations:** ^1^Child and Adolescent Psychiatry, Boston Children's Hospital, Harvard Medical School, 300 Longwood Avenue, Fegan 8, Boston, MA 02115, USA; ^2^Adult Psychiatry, New York Medical College, Valhalla, NY, USA; ^3^Adult Psychiatry, Harvard South Shore Psychiatry, Brockton, MA, USA; ^4^Duke University School of Medicine, Durham, NC, USA; ^5^Old Dominion University, Norfolk, VA, USA; ^6^Northwell Health, Zucker Hillside Hospital, Glen Oaks, NY, USA

## Abstract

Synthetic cannabinoids- (SCs-) induced psychosis is a growing public health concern. It leads to significant impairment, including emotional distress, difficulty communicating, and other debilitating symptoms. In this case report, we discuss a patient with no previous history of psychotic symptoms, presenting with first-episode psychosis in the context of progressive, acutely worsening, disorganized, psychotic thoughts and behaviors following prolonged use of SCs. We also discuss relevant literature on SCs-induced psychosis, highlighting its prevalence, presentation, diagnosis, and recommended management. It is important to diagnose and treat SCs-induced psychosis as early and efficiently as possible, in order to alleviate symptoms while limiting functional impairment and emotional distress to the patient.

## 1. Introduction

Use of synthetic cannabinoids (SCs) is a growing problem internationally. Frequency of use may be influenced by multiple factors including a desire to get high while avoiding a positive urine toxicology, the curiosity for trying a new drug, and the misconception that it is not illegal and therefore it is safe [1, 2]. One study found that, in the United States, SCs are the second most frequently used illicit substance after cannabis [3]. The majority of users are Caucasian, single men, with a mean age of 26, and adolescents account for 40% of users [2, 4–7]. It appears that groups, such as habitual cannabis users, university students, dance club attendees, and military personnel, may be the most common users of SCs [5, 6]. Approximately 84–98% of SCs users have been reported to also use cannabis [2, 5–7]. The most common route of administration is smoking via cigarettes, blunt, or pipe, but SCs can also be taken orally, rectally, or via vaporization [2, 7].

It is understood that SCs are often coconsumed as a blend or combination of a variety of different chemical compounds. There has been ongoing synthesis and development of new SCs, with 177 entities reported as new psychoactive substances to the United Nations Office of Drug and Crime (UNODC) [8, 9]. Although there are many new analogues created daily, the majority of SCs can be categorized into seven distinct structural groups, including naphthoylindoles (e.g., JWH, including subcompounds JWH-018, JWH-073, and JWH-398), naphthylmethylindoles, naphthoylpyrroles, naphthylmethylindenes, phenylacetylindoles (e.g., benzoylindoles, JWH-250), cyclohexylphenols (e.g., CP 47,497 and homologues of CP 47,497), and classical cannabinoids (e.g., HU-210) [1, 10, 11]. Most of the ingested synthetic substances contain a mixture of 2 or more of these compounds; for example, K2 or “Spice” and related drugs often contain a mixture of JWH-018 and CP-47,497 and may also contain other SCs [7, 10, 11]. Naturally occurring psychoactive plants and herbs, such as* Leonotis leonurus* (i.e., wild dagga) and* Pedicularis densiflora* (i.e., Indian warrior), are often used as carriers for the aerosolized synthetic cannabinoids [5, 7, 11].

Synthetic cannabinoids mainly interact with cannabinoid receptors (CB) [2–5, 7, 10]. One type of CB, CB1 receptors, is mainly located in the brain and throughout the central nervous system [3]. The second major type of CB, CB2 receptors, is mainly found in the peripheral nervous system, in the immune system where they mediate the immunomodulatory effects of cannabinoids, and only sparsely in the central nervous system [2]. The role of CB2 receptors in the central nervous system has yet to be elucidated [2, 12]. Similarly, SCs' effects on CB2 receptors are not completely understood. However, SCs are known to act as high potency, complete agonists at the CB1 receptor [2, 3, 5, 7, 10]. Thus SCs exert stronger effects at these receptors relative to tetrahydrocannabinol (THC), which is only a partial agonist [2, 3, 5, 7, 10]. The effect of SCs on the CB1 receptor has been reported to be five times greater than that of THC [10].

Symptoms of intoxication following use of SCs often include paranoia, psychosis (including delusional thoughts and hallucinations), and severe agitation [1, 5, 7, 13]. Some of these hallucinations differ from those more common in endogenous psychosis, with prominent visual changes such as “flashes of color,” “geometric patterns,” “fractals,” and “trails” [2]. Other psychiatric symptoms include depersonalization, dissociation, anxiety, and catatonia [2]. These effects usually begin within minutes of use and last 2–6 hours, though in some cases the effects last much longer and can range from 1 week to several months [1–3, 5, 7]. Persistent, delayed psychotomimetic effects of ingestion may resemble the trajectory and severity of psychotic symptoms seen in conditions such as schizophrenia [1].

Medical complications following ingestion of SCs can be neurological (61.9%), cardiovascular (43.5%), gastrointestinal (21.1%), respiratory (8.0%), ocular (5%), dermal (2.6%), renal (0.9%), and hematological (0.4%) complications [2]. Common neurological effects include tremor, ataxia, nystagmus, fasciculations, and hypertonicity. Seizures are less common but have also been reported [3]. The most common cardiovascular effects are tachycardia and hypertension, though fatal SCs-induced myocardial infarctions have also been reported [3]. Gastrointestinal effects include nausea, vomiting, and increased appetite [3]. Renal effects can include acute kidney injury, requiring hemodialysis in severe cases [3, 14].

Diagnosis by urine and serum toxicology is difficult because drug designers frequently change functional groups, change substitutions, and alter moieties of substances; these modifications interfere with detection on these screens [5, 7, 15]. Some military grade screens can detect the more common SCs, but detection is often not possible in a hospital setting; these limitations may be related to the use of these products by those wishing to avoid persecution and positive tests on routine hospital and institution-implemented screenings [5, 7, 10, 15]. When a detailed history with accompanying symptoms leads to strong suspicion, in addition to urine and serum toxicology, other diagnostic tests such as electrolytes, CBC, cardiac enzymes, and EKG should be used to detect any physiological complications that may be present [7, 13].

Treatment often includes supportive measures since no specific antidote is available [5, 7]. Psychiatric symptoms are usually managed with monitored observation [5, 7] and psychiatric consultation if symptoms persist. Benzodiazepines are often used to manage anxiety and agitation, while antipsychotics are second-line agents, as they may potentially lower seizure threshold (increasing risk, as SCs often lower seizure threshold) [3, 5, 7]. Long-term treatment options include substance abuse treatment programs that focus on behavioral change, individual and group counseling, cognitive behavioral therapy (CBT), and 12-step treatment groups [5, 7].

## 2. Case Report

Herein we describe an 18-year-old antipsychotic-naïve African American male with no past psychiatric history or hospitalizations, who was admitted after presenting to the psychiatric emergency room (ER) with a first-time psychotic episode in the context of prolonged synthetic cannabinoid agonist ingestion via inhalation. This patient “Mr. A” presented after experiencing over 1.5 weeks of insomnia, elated mood, agitation, paranoid ideation, beliefs that others were inserting thoughts into his head (“thought insertion”) and that his thoughts could be read by others (“thought broadcasting”), pacing, bizarre delusional thoughts with thought derailment, and disorganized behavior.

Collateral from patient's mother revealed progressive increase in substance use (particularly cannabinoids via smoke/inhalation) in the preceding 4 months, paranoid auditory hallucinations, delusions, insomnia with 0–2 hours of sleep per night, confusion, decreased short-term memory, and worsened symptoms in the previous 2 weeks, affecting his activities of daily living. She reported that 3-4 days before admission, Mr. A exhibited progressive rigidity, intermittent mutism, and cognitive slowing. Furthermore, Mr. A's mother reported that the day prior to admission, he exhibited acute confusion, amnesia, and verbalized delusions that his uncle, with whom he previously had a friendly and close relationship, was trying to kill him and that he would not be able to stay at his job because people were watching him, trying to “sell his soul” and “make his arms shorter.” On initial assessment, Mr. A's thought process was disorganized; he was confused, irritable, and avolitional, displaying mild rigidity, catatonia, and intermittent mutism, reacting to internal stimuli, mumbling, and frequently providing only simple “yes” or “no” answers. He adamantly denied any history of suicidal ideation, plan, intent, or self-inflicted physical injuries. Mr. A appeared hunched over with rigid, flexed, inwardly rotated extremities; he retained a stiff posture throughout the encounter. His eyes were initially closed, though he was awake and responding to painful stimuli. Reflexes were normal in the upper and lower extremities and there were no signs of cog-wheeling or clasp-knife rigidity.

His affect was guarded, suspicious, and perplexed with apparent slowed cognition, thought blocking, and stuttering. He appeared to be responding to auditory hallucinations, which he only vaguely endorsed following repeated questioning during the interview. The patient denied all symptoms related to mania. There was no prior history of catatonia, dystonia, stiffness, mutism, tic disorder, obsessive-compulsive disorder, or involuntary movements. Mr. A had no known drug or environmental allergies. Blood alcohol was negative, yet the urine toxicology was positive for cannabinoids. Blood labs, including N-methyl-D-aspartate (NMDA) antibodies, all were within normal limits as was his head computerized tomography scan. Magnetic resonance imaging, with sedation for control of agitation, and electroencephalography were unremarkable.

Social history revealed that Mr. A dropped out of school in 11th grade due to difficulties concentrating, consistent with his earlier educational history. This supports reported history that he did not have any disorientation or acute cognitive decline from his baseline at that time. In addition, he had spent time in a correctional facility 2 years prior to admission after violating a restraining order obtained by his mother for agitated, violent behaviors, verbal abuse, and physical abuse, which had not recurred since. There was no known family psychiatric history, yet knowledge of any psychiatric illness or substance abuse was limited on his paternal side because Mr. A had never known his father, who had no contact with the family since Mr. A's birth. Mr. A reported alcohol consumption 1-2 times per week, with an average of 1-2 mixed alcoholic beverages on each use, in social settings. He reported that, for the previous 3-4 weeks, he had only been using what he believed to be synthetic cannabinoids—smoking multiple “joints” and “blunts” with “K2” and “spice” daily, but previously he had used cannabis as well. He denied history of recent sexual encounters or any history of sexually transmitted diseases (STDs).

During the two-week hospital course, Mr. A was treated with intermittent doses of lorazepam 2 mg orally, as needed, with significant improvement in stiffness. He was stabilized over 1.5 weeks on oral risperidone, 2 mg in the morning and 3 mg at bedtime, with resolution of paranoid ideation, abnormal thought processes, and stiffness. As the risperidone was titrated, the need for lorazepam progressively decreased. When mood was stable, with resolved psychosis and paranoia, Mr. A was discharged and followed as an outpatient. On the first postdischarge assessment (1 week later), Mr. A was coherent, with logical thought process, though he displayed mild bilateral stiffness, stuttering, and gait abnormalities with short-stepping, along with markedly constricted affect—all suggestive of mild antipsychotic-induced extrapyramidal symptoms (EPS). The oral risperidone dose was tapered to 2 mg twice daily (BID) and oral benztropine 0.5 mg at bedtime was added with improvement in affect, dystonia, and continued mood stability. One week later, risperidone was tapered to 3 mg at bedtime, with continued improvement. Mr. A was no longer psychotic or disorganized on any assessments since admission to the outpatient clinic, and the dystonia, EPS, and stiffness were markedly improving.

At the next appointment, 2 weeks after discharge, Mr. A presented with recurrence of catatonia, stiffness, mutism, auditory hallucinations, disorganized speech and thought process, and blunted affect. He confirmed that he had relapsed to using SCs and reported noncompliance with his risperidone for the last 4 days. Mr. A again exhibited pacing, along with bizarre behavior and delusions, stating that “security guards” at the vocational job location could read his thoughts and were tracking him. This acute worsening led to rehospitalization for stabilization and management. On hospitalization, with discontinuation of SCs use and reinitiation of risperidone, symptoms again improved drastically, and risperidone was tapered to 2 mg BID. Mr. A was again discharged for further follow-up, including a partial hospitalization program at a combined substance abuse and psychiatric treatment (“dual-diagnosis”) center with daily activities and structured treatment. Mr. A continued to do well on this regimen.

## 3. Discussion

Our patient experienced a first-episode of psychosis related to use of SCs, with disorganized and paranoid thought processes. Similar symptoms have been reported in many patients with preexisting psychotic conditions after ingestion of SCs [16]. Additionally, cases of first-episode psychosis have been reported in the context of acute and prolonged ingestion of SCs [2]. Cases reported include a young student experiencing anxiety, paranoia, and visual and auditory hallucinations after use of SCs for three weeks [17] and a 36-year-old male that exhibited odd behavior and irritability for two weeks after using SCs for four consecutive weeks. His symptoms resolved when he stopped using SCs [18]. One case-series included 10 male subjects, aged 21 through 25, without prior psychiatric history (though 1 had a family history of psychosis), who developed psychotic symptoms after prolonged use of SCs and were hospitalized on a psychiatric unit [16]. These patients' symptoms included paranoia, thought disorders, and suicidal ideation [16]. In this case-series, 7 of the 10 patients received antipsychotic treatment, and hospitalizations ranged from 6 to 10 days. In 7 of the patients, psychosis resolved between 5 and 8 days after admission, while 3 patients had psychosis that lasted for more than 5 months [16]. Symptoms including a distinct period of intermittent, stuporous appearance were common, along with odd or flat affect, thought blocking, disorganized speech and behavior, auditory hallucinations, visual hallucinations, paranoid delusions, and alogia [16]. Suicidal ideation and insomnia were also reported [16]. As discussed above, the fact that SCs are often mixed with other psychoactive drugs of abuse, including cannabis, can complicate or confound the diagnosis of SCs-related psychosis. Our case is particularly notable for strong response to antipsychotic medication, subsequent relapse of psychotic symptoms following relapse in use of SCs, and repeated response to a second round of antipsychotic medication treatment.

Various studies have suggested that use of SCs may be associated with development of psychotic symptoms [10, 16, 19, 20]. Whether many patients who develop first-episode psychosis after ingestion of SCs have an underlying predisposition to psychosis [1, 21] warrants further investigation.

In our case, psychosis due to ingestion of SCs was diagnosed efficiently, which facilitated successful treatment, beginning with lorazepam administration for catatonic symptoms and with risperidone to target psychotic symptoms. The strong response to risperidone both as an initial treatment and following relapse provides additional information indicating that clinicians can use a previously effective antipsychotic for relapse of SCs-induced psychosis.

Clinicians should be aware of the possibility of cannabinoid-induced psychotic delusions, paranoia, and distorted thoughts after recent ingestion of SCs, in order to optimally identify and treat this condition. Treatment may be most effective when including inpatient admission, maintaining a substance-free period, and medications for psychosis.
